# Highly efficient hybridoma generation and screening strategy for anti-PD-1 monoclonal antibody development

**DOI:** 10.1038/s41598-022-20560-6

**Published:** 2022-10-22

**Authors:** Tanapati Phakham, Chatikorn Boonkrai, Tossapon Wongtangprasert, Thittaya Audomsun, Chadaporn Attakitbancha, Pijitra Saelao, Phijitra Muanwien, Sarintip Sooksai, Nattiya Hirankarn, Trairak Pisitkun

**Affiliations:** 1 Interdisciplinary Program of Biomedical Sciences, Graduate School, Chulalongkorn University, Bangkok, Thailand; 2 Center of Excellence in Systems Biology, Faculty of Medicine, Chulalongkorn University, Bangkok, Thailand; 3 The Excellence Chulalongkorn Comprehensive Cancer Center, King Chulalongkorn Memorial Hospital, Bangkok, Thailand; 4 The Institute of Biotechnology and Genetic Engineering, Chulalongkorn University, Bangkok, Thailand; 5 Cancer Immunotherapy Excellence Center, Faculty of Medicine, Chulalongkorn University, Bangkok, Thailand; 6 Center of Excellence in Immunology and Immune Mediated Diseases, Department of Microbiology, Faculty of Medicine, Chulalongkorn University, Bangkok, Thailand

**Keywords:** Biotechnology, Drug discovery

## Abstract

Programmed cell death protein 1 (PD-1) plays a significant role in suppressing antitumor immune responses. Cancer treatment with immune checkpoint inhibitors (ICIs) targeting PD-1 has been approved to treat numerous cancers and is the backbone of cancer immunotherapy. Anti-PD-1 molecule is necessary for next-generation cancer immunotherapy to further improve clinical efficacy and safety as well as integrate into novel treatment combinations or platforms. We developed a highly efficient hybridoma generation and screening strategy to generate high-potency chimeric anti-PD-1 molecules. Using this strategy, we successfully generated several mouse hybridoma and mouse/human chimeric clones that produced high-affinity antibodies against human PD-1 with high-quality in vitro PD-1/PD-L1 binding blockade and T cell activation activities. The lead chimeric prototypes exhibited overall in vitro performance comparable to commercially available anti-PD-1 antibodies and could be qualified as promising therapeutic candidates for further development toward immuno-oncology applications.

## Introduction

Cancer has become a leading cause of death globally for several decades, and its incidence still increases yearly. Cancer immunotherapy has recently become a standard cancer treatment strategy by harnessing a patient's immune system to battle cancer^1,2^. Immune checkpoint inhibitors (ICIs), mainly in the monoclonal antibody (mAb) format targeting immune checkpoint molecules, are currently the most efficacious cancer immunotherapy agents^3,4^. Among a couple of FDA-approved ICIs, programmed cell death protein 1 (PD-1) inhibitors obtain the blockbuster status based on the current and forecasting number of approved indications for cancer treatment^5,6^. In particular, the U.S. FDA-approved anti-PD-1 monoclonal antibodies pembrolizumab (KEYTRUDA) and nivolumab (OPDIVO) earned more than 19.1 Billion USD in 2019, and their sales are still rising annually because of their outstanding clinical response^7^. Although the approach using these agents as monotherapy has exhibited remarkable antitumor efficacy, further clinical improvement has been demonstrated using a combination with different ICIs or other treatment modalities^8,9^. Hence, the development of effective combination therapy strategies for cancer immunotherapy is the current trend in the immuno-oncology research field^10^. Moreover, novel ICIs in the forms of bispecific antibodies (BsAbs), dual-affinity retargeting proteins (DART), nanobodies (Nb), and scFvs secreted by CAR-T cells are being developed to augment the clinical response in cancer immunotherapy^11–13^. Notably, ICIs targeting PD-1 are commonly used as backbones for these combinatorial and innovative antibody therapies. Several anti-PD-1-based combinations have been approved (with anti-CTLA-4)^5,14^ or under various stages of investigation (e.g., with anti-CD137^15^, anti-LAG-3^16^, or tyrosine kinase inhibitors^17^). Additionally, encouraging preclinical and clinical data have been reported on anti-PD-1-based bispecific molecules such as anti-PD-1/anti-HER2 bispecific IgG-scFv^18^, anti-PD-1/anti-EGFR bispecific IgG-scFv^19^, and anti-PD-1/anti-CTLA-4 bispecific IgG^20^. Thus, anti-PD-1 has been regarded as a cornerstone of current cancer immunotherapy and is expected to maintain its essential role in the future quest of cancer medicine^21,22^. Therefore, the ongoing development of anti-PD-1 molecules is necessary for next-generation cancer immunotherapy to maximize clinical efficacy and avoid intellectual property conflicts^23,24^.

This work aims to generate a high-potency chimeric anti-PD-1 mAb using a highly efficient hybridoma generation and screening strategy. We successfully generated several mouse hybridoma and mouse/human chimeric clones that produced high-affinity antibodies against human PD-1 with high-quality in vitro PD-1/PD-L1 binding blockade and T cell activation activities. The lead chimeric clone exhibited in vitro checkpoint inhibitory performance comparable to commercially available anti-PD-1 antibodies. Accordingly, these clones could be qualified as potential candidates for further development as either single, combination, or innovative cancer immunotherapies.

## Results

**Figure 1:**
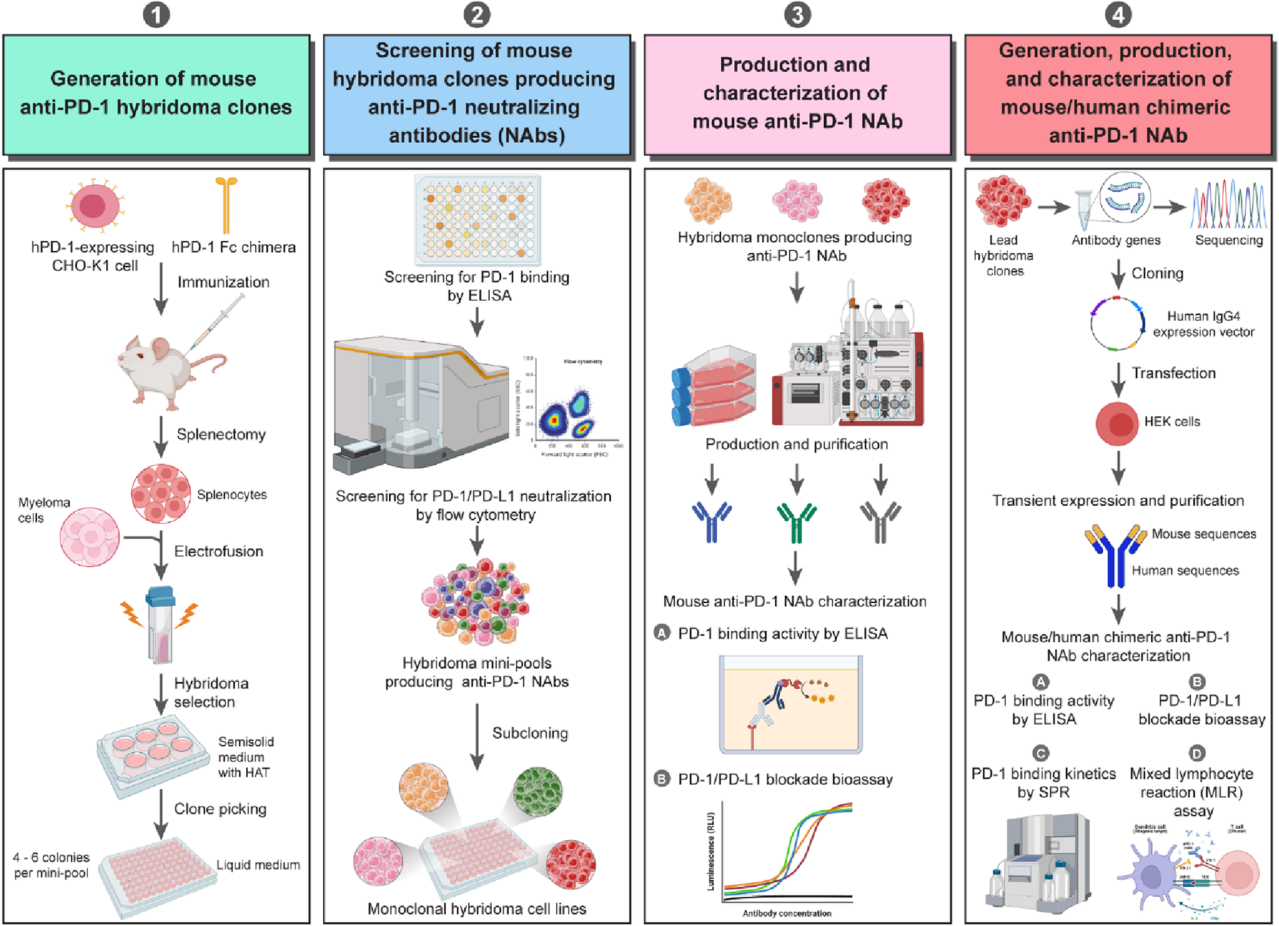
Workflow of the generation, screening, and characterization of the chimeric anti-PD-1 monoclonal antibodies. Four sequential steps of this workflow are displayed.

In this study, we demonstrated a highly efficient strategy for generating, screening, and characterizing chimeric anti-PD-1 antibodies; see the workflow in Fig. 1. This workflow comprises 4 major steps, including (1) generation of mouse anti-PD-1 hybridoma clones, (2) screening of mouse hybridoma clones producing anti-PD-1 neutralizing antibodies (NAbs); inhibit the PD-1 and PD-L1 interaction, (3) production and characterization of mouse anti-PD-1 NAbs, and (4) generation, production, and characterization of mouse/human chimeric anti-PD-1 NAbs. The details of this workflow will be described in the following sections.

### Generation of hyperimmunized mice to hPD-1

**Figure 2:**
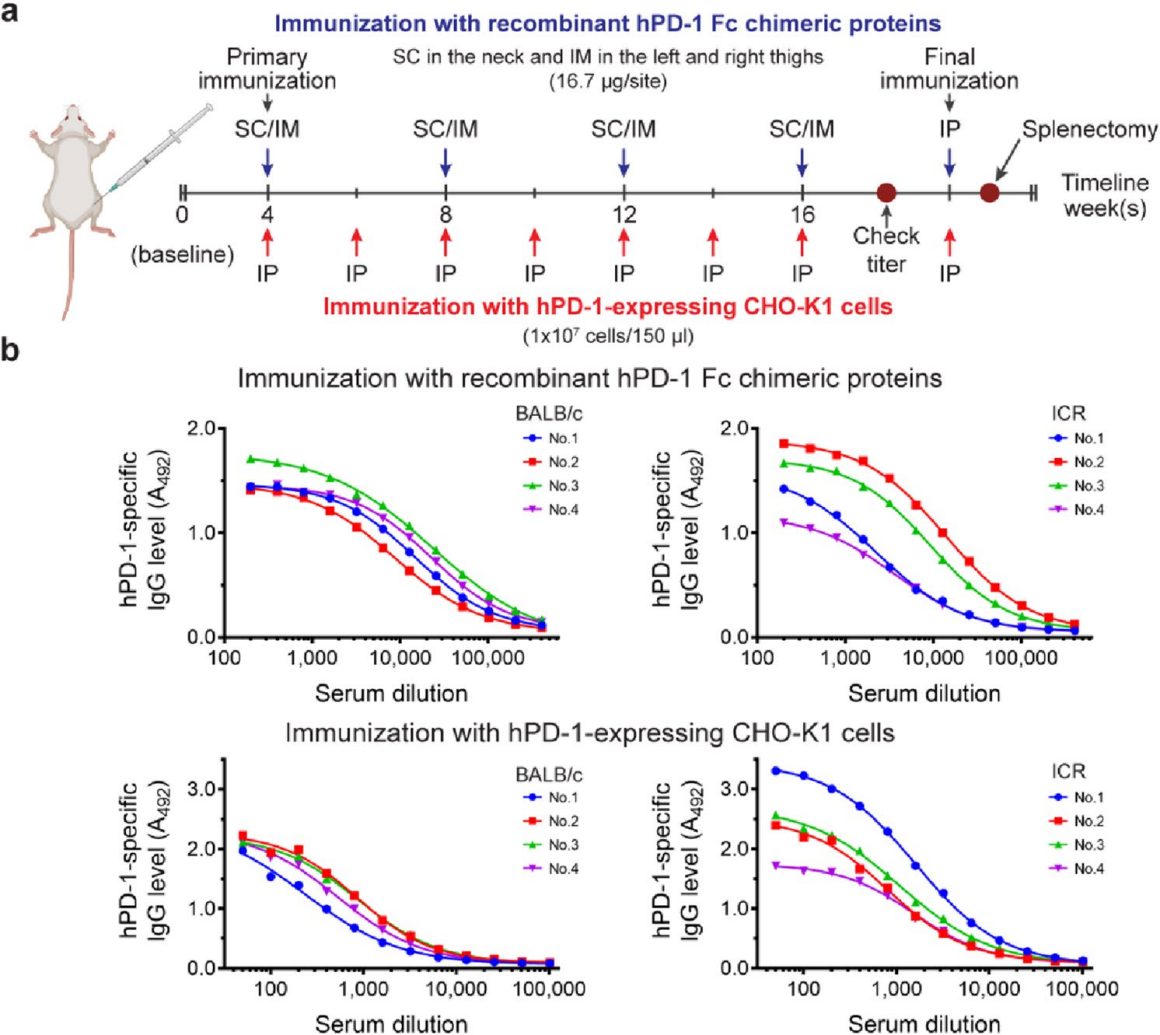
Mouse immunization strategy and hPD-1-specific IgG titration curves. **(a)** Immunization timeline. BALB/c and ICR mice were immunized with either recombinant hPD-1 Fc chimeric proteins or hPD-1-expressing CHO-K1 cells. Immunogens were injected via multiple routes, including SC; subcutaneous, IM; intramuscular, and IP; intraperitoneal. **(b)** hPD-1-specific mouse IgG titration curves. Blood was collected 4 days after 16 weeks of immunization (check titer). Serial dilutions of each mouse serum were added to the hPD-1 His tag-coated ELISA plates. Goat anti-mouse IgG Fc-γ-HRP was used to detect PD-1-specific mouse IgG levels. The mouse with the highest IgG titer (at the lowest dilution) from each immunogen was selected for final immunization, followed by splenectomy for hybridoma generation.

Our immunization strategy employed two forms of immunogens administered via multiple routes and two strains of mice to increase the chance of activating highly potent PD-1 neutralizing B cell clones. BALB/c or ICR mice (*n* = 4 mice per strain) were immunized with either recombinant hPD-1 Fc chimeric proteins (protein group or P) with Freund's adjuvant via SC, IM, and IP or hPD-1-expressing CHO-K1 cells (cell group or C) via IP (see Fig. 2a).

All mice successfully generated hPD-1-specific IgG titers on day seven after the 16-week immunization course, as shown in Fig. 2b (all preimmunization IgG titers were undetectable, data not shown). With both immunogens, ICR mice produced a higher variation in the hPD-1-specific IgG titration curves than BALB/c mice (coefficient of variation of A_492_ values of BALB/c versus ICR at the lowest serum dilution of the protein group were 9.46 versus 21.71, respectively and that of the cell group were 4.97 versus 26.23, respectively). Based on these results, only two ICR mice producing the highest titers, No. 2 from the protein group and No. 1 from the cell group, were selected for the final immunization. Four days later, the mice were anesthetized with isoflurane in an isoflurane-saturated euthanasia chamber. Subsequently, their spleens were aseptically harvested for hybridoma generation.

### Efficient screening and selection strategy for hybridoma clones producing anti-PD-1 antibodies with PD-1/PD-L1 binding blockade (neutralizing) activity

**Figure 3:**
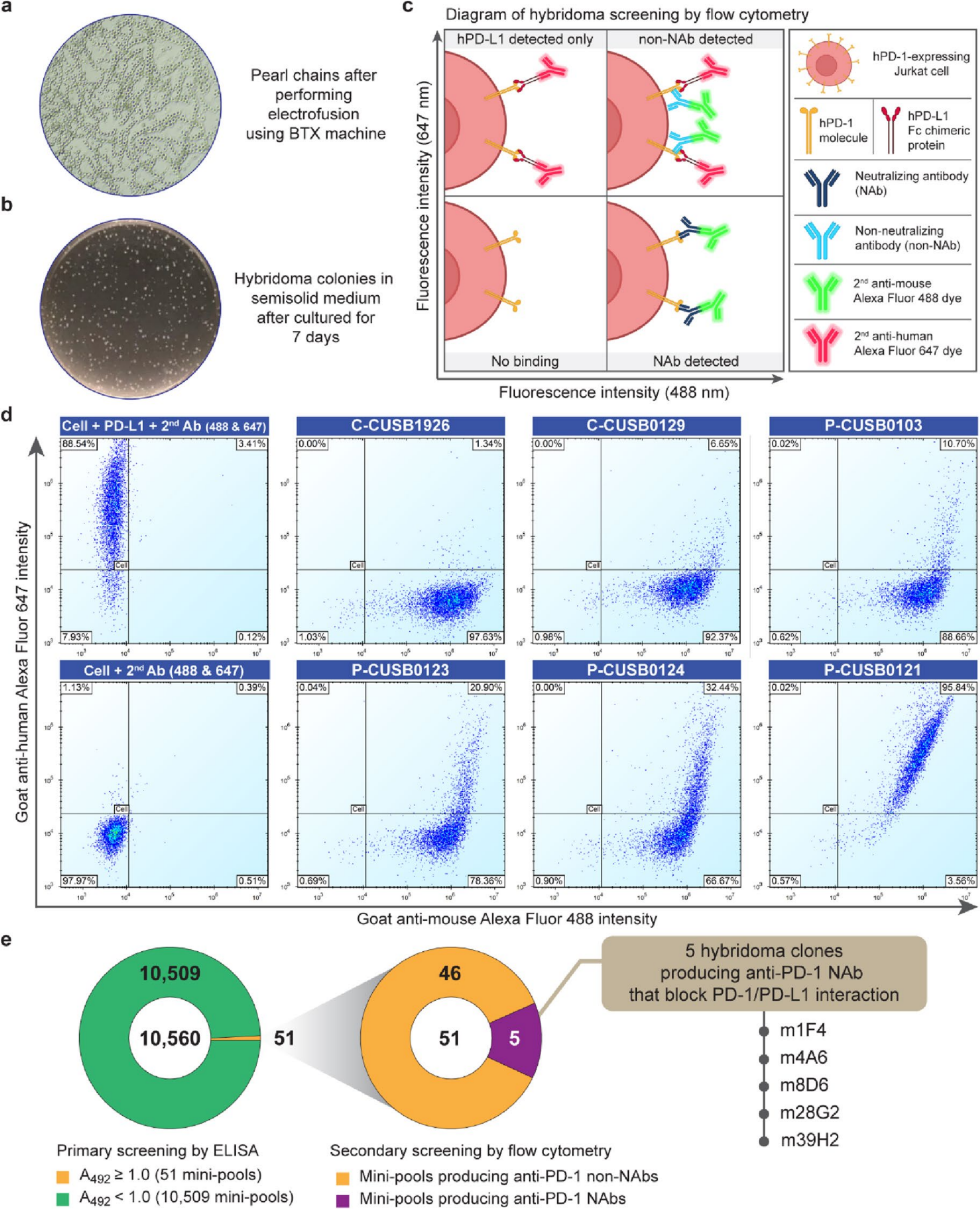
Screening and selection strategy for hybridoma clones producing anti-PD-1 antibodies with PD-1/PD-L1 binding blockade activity. **(a)** Pearl chains of splenocyte-myeloma cells after electrofusion. **(b)** Hybridoma colonies in semisolid HAT medium after selection for 7 days. **(c)** Illustration of the anti-PD-1 NAb screening strategy using high-throughput flow cytometry. The lower left quarter (no binding) displayed no fluorescence signals detected. The upper-left quarter (hPD-L1 detected only) displayed the detection of fluorescence 647-labeled secondary antibody that bound to the hPD-L1 human Fc chimera proteins. The upper-right quarter (non-NAb detected) displayed the detection of both 647-labeled and 488-labeled fluorescence signals that bound to mouse anti-PD-1 antibody and hPD-L1 Fc chimeric protein. The lower-right quarter (NAb detected) displayed only the detection of fluorescence 488-labeled secondary antibody that bound to mouse anti-PD-1 antibody with PD-1/PD-L1 binding blockade activity. **(d)** The dot-plot results of selected hybridoma mini-pools producing anti-PD-1 antibody with various properties. The percentage of the NAb detected population is indicated in the lower-right corner. **(e)** Summary results of hybridoma clones derived from each screening step. Altogether, of these efficient strategies, we successfully isolated 5 hybridoma clones with high PD-1 binding and PD-1/PD-L1 binding blockade activities.

We carried out hybridoma generation using cell electrofusion technology, viz., a simple, fast, controllable, and reproducible technique that can obtain higher fusion efficiency than the polyethylene glycol (PEG) fusion technique^25,26^. To enhance cloning efficiency and prevent the loss of high-producing clones, a methylcellulose-based semisolid medium was chosen for hybridoma selection and cloning. Figure 3a shows the formation of splenocyte-myeloma pearl chains after electrofusion, indicating that the electrofusion was well performed. Numerous hybridoma colonies were then observed by the naked eye within 7 days after being cultured in a semisolid medium containing HAT (Fig. 3b). These results confirmed that we successfully generated hybridoma clones using the electrofusion technique. Afterward, all hybridoma colonies were randomly picked, and 6–8 clones were combined into a mini-pool and cultured in liquid medium.

We devised a highly efficient mini-pool screening strategy to minimize laborious steps and time for identifying mini-pools that generate functioning antibodies with high PD-1/PD-L1 binding blockade activities. First, mini-pools containing hybridoma clones producing hPD-1-specific IgG were screened by ELISA. From a total of 10,560 mini-pools, 51 mini-pools exhibited a high PD-1 binding signal (A_492_ > 1.0). Second, a duplex high-throughput flow cytometry-based neutralization assay was set up to rapidly distinguish hPD-1-specific neutralizing antibodies from their nonneutralizing counterparts (Fig. 3c). Using cell-based screening by high-throughput flow cytometry to directly screen for neutralizing antibodies recognizing the native conformation of the PD-1 proteins expressed on the cell surface allowed us to rapidly discover candidate high-performance clones. Briefly, the culture supernatant of each hybridoma mini-pool was added to hPD-1-expressing Jurkat cells in the presence of recombinant hPD-L1 Fc chimeric protein. Two different fluorescence-labeled secondary antibodies against either mouse Fc or human Fc were used for duplex detection. Thus, four possible outcomes were expected from this assay, i.e., no binding, hPD-L1 detected only, non-NAb detected, and NAb detected. A mini-pool that yielded more than 50% NAb detected population was considered to have a significant PD-1/PD-L1 binding blockade activity. The results demonstrated that out of 51 hybridoma mini-pools, only 5 could block the PD-1/PD-L1 interaction (Fig. 3d), where C and P indicate the mini-pool derived from mice immunized with cells and proteins, respectively. The C-CUSB0126 and C-CUSB0129 mini-pools exhibited high PD-1/PD-L1 binding blockade activity, with 97.63% and 92.37% NAb detected populations, respectively, followed by P-CUSB0103 (88.66%) and P-CUSB0123 (78.36%). The P-CUSB0124 mini-pool showed partial PD-1/PD-L1 binding blockade activity with a 66.67% NAb detected population. On the other hand, the P-CUSB0121 mini-pool is an example of mini-pools that mainly manifest nonneutralizing activity. Therefore, this strategy successfully screened for hybridoma mini-pools producing anti-PD-1 mAb with high PD-1/PD-L1 binding blockade activities against the correct protein conformation using minimal time and labor. Figure 3e summarizes data from mouse hybridoma screening. Altogether, using these screening strategies, we successfully isolated 5 mouse hybridoma mini-pools producing anti-PD-1 NAb. Consequently, 5 hybridoma mini-pools were subcloned using semisolid medium to isolate hybridoma monoclones. These isolated mouse hybridoma clones were expanded and cultured in serum-free medium for anti-PD-1 production, followed by purification.

### Functional characterization of mouse anti-PD-1 monoclonal antibodies

**Figure 4:**
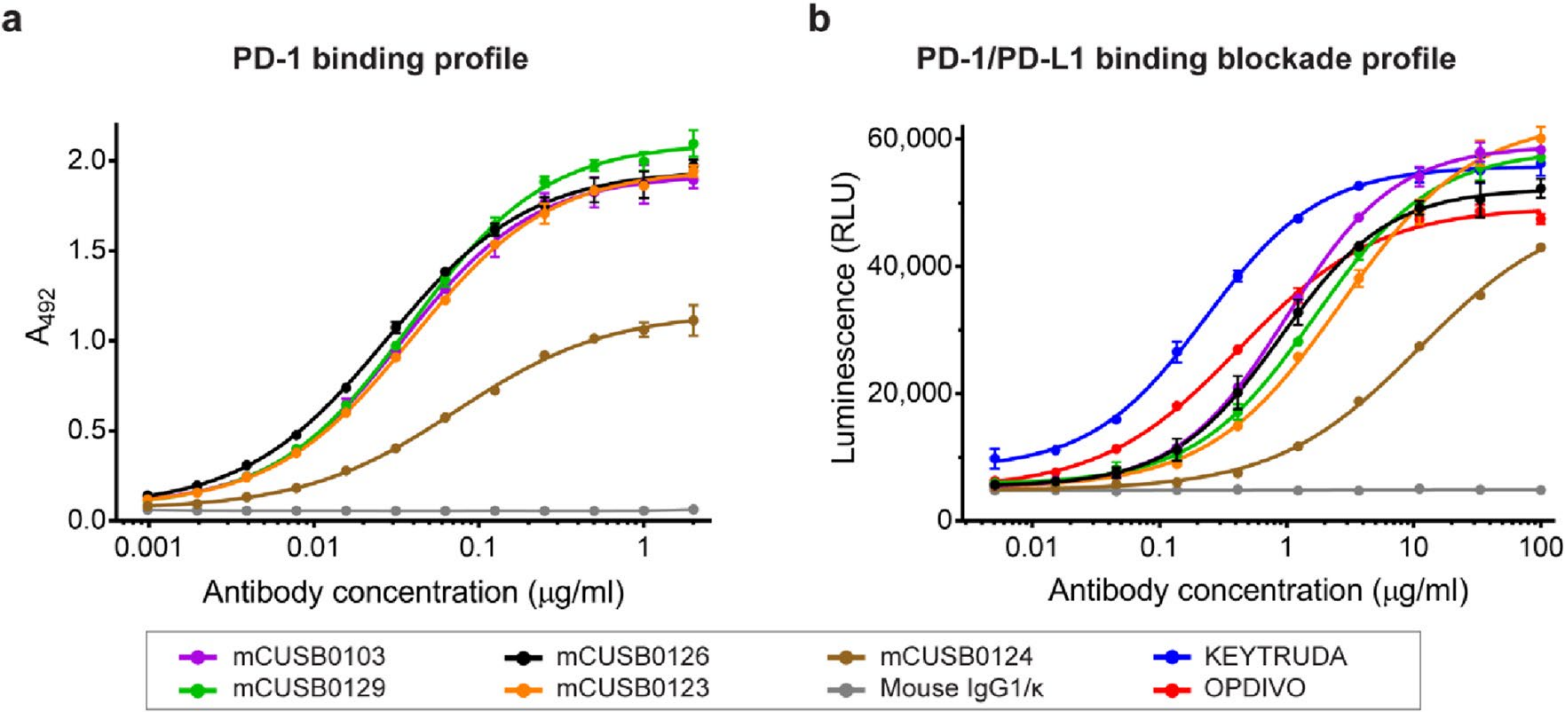
PD-1 binding and PD-1/PD-L1 binding blockade profiles of mouse anti-PD-1 monoclonal antibodies. **(a)** PD-1 binding profile of selected mouse anti-PD-1 mAbs assessed by ELISA. Serial dilutions of each purified mouse anti-PD-1 mAb were added to the hPD-1 His tag-coated ELISA plate. HRP-conjugated goat anti-mouse IgG Fc-γ was used for detection. The absorbance at 492 nm was measured. Data from triplicates are presented as the mean ± SD. **(b)** PD-1/PD-L1 binding blockade profile of selected mouse anti-PD-1 mAbs assessed by the PD-1/PD-L1 blockade bioassay. Serial dilutions of each purified mouse anti-PD-1 mAb and hPD-1-expressing Jurkat cell were added to an assay plate harboring precultured PD-L1-expressing CHO-K1 cells. After coculture for 6 h, Bio-Glo was added, and the luminescence signals were measured. The results from triplicates are presented as a relative light unit (RLU) as the mean ± SD. Commercially available anti-PD-1 mAbs, KEYTRUDA and OPDIVO, were used as positive controls, while human IgG4/κ was used as a negative control.

To characterize the functional activity of purified mouse anti-PD-1 mAbs, ELISA was used to determine the PD-1 binding activity. Figure 4a shows the PD-1 binding profile of each mouse anti-PD-1 mAb. This result showed that 4 mouse anti-PD-1 mAbs, mCUSB0103, mCUSB0126, mCUSB0129, and mCUSB0123, showed high hPD-1 binding activity with a similar profile, while mCUSB0124 displayed lower PD-1 binding activity.

Furthermore, the PD-1/PD-L1 binding blockade activity of these purified mouse anti-PD-1 mAbs was evaluated using the PD-1/PD-L1 blockade bioassay. Our results showed that all mAbs could inhibit the interaction between PD-1 and PD-L1, as shown in Fig. 4b. The mCUSB0126, mCUSB0103, mCUSB0129, and mCUSB0124 mAbs exhibited high PD-1/PD-L1 binding blockade activity, with IC_50_ values of 0.891 µg/ml, 1.005 µg/ml, 1.651 µg/ml, and 2.678 µg/ml, respectively, while mCUSB0124 had the lowest PD-1/PD-L1 binding blockade activity (IC_50_ = 11.170 µg/ml). This finding is related to the flow cytometry results. These results revealed that 4 mouse anti-PD-1 mAbs have high PD-1 binding activity and can inhibit the interaction between PD-1 and PD-L1 with low IC_50_ values. In addition, these purified mouse anti-PD-1 mAbs exhibited high PD-1 binding affinity in the subnanomolar range (Table 1).

**Table 1:** Functional properties of mouse anti-PD-1 monoclonal antibodies.

Mouse antibodies	PD-1 binding activity, EC_50_ (ng/ml)	PD-1/PD-L1 binding blockade activity, IC_50_ (ng/ml)	Binding kinetics		
			*k*_*on*_ (1/Ms)	*k*_*off*_ (1/s)	*K*_*D*_ (nM)
mCUSB0103	34.42	1,005	3.52E + 05	5.45E-04	1.549
mCUSB0123	39.23	2,678	3.68E + 05	5.41E-04	1.468
mCUSB0124	73.08	11,170	9.37E + 04	1.67E-03	1.784
mCUSB0126	27.83	891	3.28E + 05	6.09E-04	1.855
mCUSB0129	38.51	1,651	3.62E + 05	3.46E-04	0.954

### Generation and functional characterizations of chimeric anti-PD-1 monoclonal antibodies

According to human safety issues, these mouse mAbs cannot be used as therapeutic antibodies in humans because they can cause immunogenicity. Accordingly, chimeric mAbs were constructed by grafting mouse variable regions to human IgG4/kappa constant regions and cloned into the pcDNA3.4 expression vector. Chimeric anti-PD-1 mAbs were transiently expressed in HEK293 cells for seven days and purified using protein A chromatography. Then, purified chimeric anti-PD-1 mAbs were characterized to confirm their biological functions.

**Figure 5:**
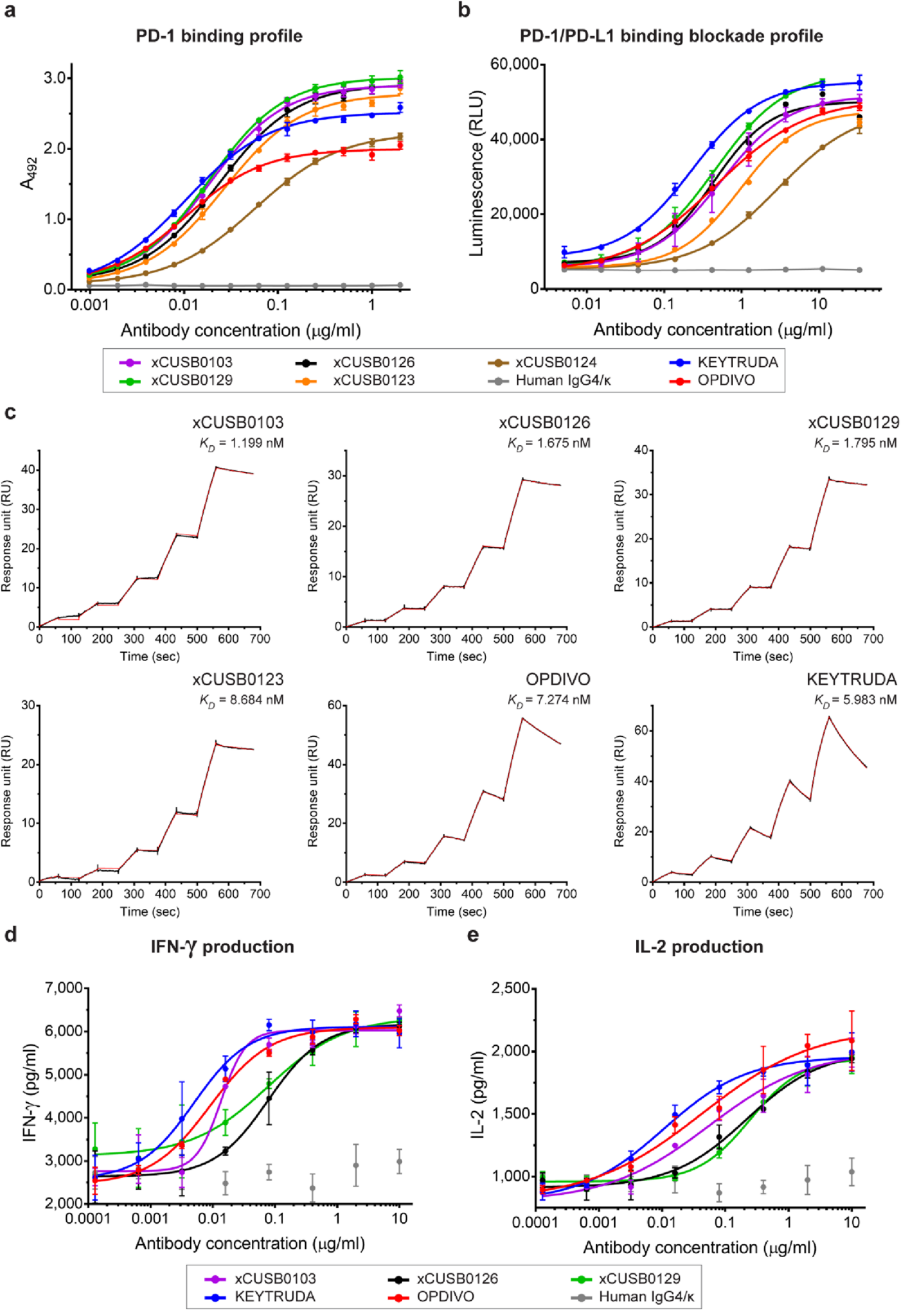
Functional characterization of chimeric anti-PD-1 monoclonal antibodies. **(a)** PD-1 binding profile of generated chimeric anti-PD-1 mAbs. ELISA was used to determine the binding activity. Data from triplicates are presented as the mean ± SD. **(b)** PD-1/PD-L1 binding blockade profile of generated chimeric anti-PD-1 mAbs. The PD-1/PD-L1 blockade bioassay was used to determine the PD-1/PD-L1 binding blockade activity. The results from triplicates are presented as RLUs as the mean ± SD. **(c)** Sensorgrams of the binding kinetics between the generated chimeric anti-PD-1 mAbs and the hPD-1 His tag assessed by SPR. Single-cycle kinetics were performed by sequentially injecting the hPD-1 His tag at various concentrations to capture chimeric mAb on a protein G sensor chip. The *K*_*D*_ value was calculated using a 1:1 Langmuir model fitting curve and presented in the sensorgram. The potency of the generated chimeric anti-PD-1 mAbs on T cell activation was evaluated by the MLR assay. Serial dilutions of each chimeric mAb and antigen-presenting cells were coincubated with the effector cells. After coculture for 3 days, the secretion levels of **(d)** interferon gamma (IFN-γ) and **(e)** interleukin 2 (IL-2) were quantified. Data from triplicates are presented as the mean ± SD. Commercially available anti-PD-1 mAbs, KEYTRUDA and OPDIVO, were used as positive controls, while human IgG4/κ was used as a negative control in all experiments.

ELISA was used to confirm the PD-1 binding activity. Serial dilutions of anti-PD-1 mAbs were added to hPD-1-coated ELISA plates. The results revealed that the xCUSB0103, xCUSB0129, xCUSB0126, and xCUSB0123 chimeric mAbs showed high PD-1 binding activity compared to commercial anti-PD-1 antibodies (Fig. 5a). However, the xCUSB0124 chimeric mAb shows lower binding activity than the others. In contrast, the human IgG4 isotype control did not exhibit any binding activity to hPD-1.

Furthermore, the PD-1/PD-L1 binding blockade activity of all chimeric mAbs was determined. Figure 5b reveals that the xCUSB0126, xCUSB0129, and xCUSB0103 chimeric mAbs exhibit high PD-1/PD-L1 binding blockade activity with IC_50_ values of 429.6 ng/ml, 454.8 ng/ml, and 539.4 ng/ml, respectively, in a similar dose-dependent manner compared with the commercial anti-PD-1 mAb (OPDIVO, IC_50_ = 460.9 ng/ml). However, the xCUSB0123 and xCUSB0124 chimeric mAbs showed lower PD-1/PD-L1 binding blockade activity than the others.

Binding kinetics is another essential factor for therapeutic mAbs. Hence, all chimeric anti-PD-1 mAbs were also assessed for binding kinetics by SPR to determine the association rate and dissociation rate between the mAb and target molecule. Figure 5c shows a single-cycle SPR sensorgram of chimeric mAbs and commercial anti-PD-1 mAbs (KEYTRUDA and OPDIVO) to the hPD-1 His tag. The results showed that the 3 chimeric mAbs had higher PD-1 binding kinetics than the commercial anti-PD-1 mAbs. The xCUSB0103 chimeric mAb has the highest binding affinity (*K*_*D*_ = 1.199 nM), followed by the xCUSB0126 chimeric mAb (*K*_*D*_ = 1.675 nM) and xCUSB0129 chimeric mAb (*K*_*D*_ = 1.795 nM), while the *K*_*D*_ values of KEYTRUDA and OPDIVO were 5.983 nM and 7.274 nM, respectively. According to the PD-1 binding and PD-1/PD-L1 binding blockade activity results, the xCUSB0124 chimeric mAb exhibited the lowest binding affinity to human PD-1 protein (data not shown). These results demonstrated that the top 3 chimeric anti-PD-1 mAbs, including xCUSB0103, xCUSB0126, and xCUSB0129, presented high PD-1 binding and PD-1/PD-L1 binding blockade activities in a similar profile to commercial anti-PD-1 antibodies. Moreover, these chimeric mAb candidates exhibited a higher affinity against hPD-1 than commercial anti-PD-1 antibodies.

Reactivation of exhausted T cells is key in cancer immunotherapy. Consequently, many ICIs target negative regulatory molecules on T cells, resulting in anergic T cells becoming activated. Hence, the MLR assay was performed to assess the potency of chimeric anti-PD-1 mAbs on T cell activation. Serial dilutions of anti-PD-1 mAbs were added to CD4^+^ T cells and dendritic cells (DCs) and then cocultured for 3 days. The secretion levels of the activating cytokines interferon-gamma (IFN-γ) and interleukin-2 (IL-2) were determined by ELISA. The MLR results via IFN-γ production (Fig. 5d) illustrated that the xCUSB0103 chimeric mAb could activate human T cells by enhancing the secretion levels of IFN-γ with an EC_50_ of 13.60 ng/ml, followed by the xCUSB0126 chimeric mAb (77.76 ng/ml) and xCUSB0129 chimeric mAb (78.05 ng/ml). In comparison, the EC_50_ values of KEYTRUDA and OPDIVO on IFN-γ secretion were 5.04 ng/ml and 9.14 ng/ml, respectively.

In addition, Fig. 5e shows that the xCUSB0103 chimeric mAb also showed a higher ability to stimulate the IL-2 production level (EC_50_, 55.71 ng/ml) than the xCUSB0126 chimeric mAb (EC_50_, 219.1 ng/ml) and xCUSB0129 chimeric mAb (EC_50_, 244.6 ng/ml). KEYTRUDA and OPDIVO stimulated the production of IL-2 with EC_50_ values of 10.93 ng/ml and 44.27 ng/ml, respectively. As expected, the human IgG4 antibody could not significantly activate T cells according to the MLR assay. These results indicated that the xCUSB0103 chimeric mAb could stimulate T cell responses in a comparable dose–response curve with a commercial anti-PD-1 antibody, OPDIVO. The functional properties of chimeric and commercial anti-PD-1 antibodies are summarized in Table 2.

**Table 2:** Functional properties of chimeric anti-PD-1 monoclonal antibodies.

Chimeric antibodies	PD-1 binding activity, EC_50_ (ng/ml)	PD-1/PD-L1 binding blockade activity, IC_50_ (ng/ml)	Binding kinetics			T cell activation	
			*k*_*on*_ (1/Ms)	*k*_*off*_ (1/s)	*K*_*D*_ (nM)	IFN-γ, EC_50_ (ng/ml)	IL-2, EC_50_ (ng/ml)
xCUSB0103	18.90	593.4	6.53E + 05	2.92E−04	0.448	13.6	55.7
xCUSB0123	26.59	956.7	2.98E + 05	3.66E−04	1.230	N/A	N/A
xCUSB0124	56.98	2,929	1.54E + 05	9.12E−04	5.920	N/A	N/A
xCUSB0126	23.71	429.6	4.93E + 05	2.85E−04	0.577	77.7	219.1
xCUSB0129	18.67	454.8	4.71E + 05	2.93E−04	0.621	78.0	244.6
KEYTRUDA	10.33	227.7	6.71E + 05	4.019E−3	5.983	5.0	10.9
OPDIVO	10.57	460.9	1.92E + 05	1.397E−3	7.274	9.1	44.2

*N/A* Not available.

## Discussion

The present study demonstrates the details of the hybridoma generation, selection, and screening procedure to successfully develop high-potency chimeric anti-PD-1 mAbs for cancer immunotherapy. The top 5 mouse mAb candidates revealed that they strongly bind to human PD-1 and effectively inhibit the interaction between PD-1 and PD-L1. In addition, chimerization of these antibodies was performed. The generated chimeric mAbs were also characterized for their functional properties. The results showed that the xCUSB0103, xCUSB0126, and xCUSB0129 chimeric mAbs exhibited high PD-1 binding activity and high PD-1/PD-L1 binding blockade activity comparable to commercial anti-PD-1 mAb, OPDIVO. These chimeric mAb candidates displayed higher PD-1 binding affinity than commercial anti-PD-1 antibodies, both KEYTRUDA and OPDIVO. Interestingly, the xCUSB0103 chimeric mAb displayed high potency on T cell activation via IFN-γ and IL-2 release in vitro.

The mouse immunization strategy plays a role in generating mouse immune responses to foreign antigens. To generate hyperimmunized mice, immunogens are usually prepared in Freund's adjuvants, which could be incomplete Freund's (without mycobacteria component) or complete Freund's (with inactivated and dried mycobacteria), in the form of a water-in-oil emulsion. This study used Adjulite Freund's adjuvants (both CFA and IFA) in protein antigen preparation to obtain a high antibody titer response in mice related to a previous report^27^. In addition, cells overexpressing protein antigen, which provides natural posttranslational modifications with correct conformation folding, were also used for mouse immunization to increase the chance of acquiring more B cell clones targeting the correct protein conformation^28^. In addition, another essential key to success is the antigen administration routes. Hence, an efficient immunization strategy by injecting immunogens via multiple routes (e.g., IM, IP, and SC) was utilized in this study to stimulate humoral and cell-mediated immune responses^29,30^. Our results suggested that this strategy could enhance humoral immune responses, especially by stimulating B cell clones that specifically bind to the target antigen.

Another vital key in hybridoma generation is the fusion technique. Generally, the polyethylene glycol (PEG)-mediated cell fusion technique was used because it is simple and inexpensive. However, the fusion efficiency using PEG is quite low compared to another efficient technique, electrofusion^26^. The fusion technique and the selection strategy are also essential to obtaining high-quality hybridoma. Hence, this study combined the efficient electrofusion technique to maximize the fusion efficiency with effective hybridoma selection using semisolid medium to obtain numerous antigen-specific producing clones. Our finding demonstrated that the combination of fusion and selection strategy provided more than 10,500 mini-pools, and 51 mini-pools (0.48% of highly efficient clones) exhibited a high binding activity to their target, which is consistent with a previous report^31^. This efficient screening strategy has been reported to screen antibodies directed against antigens expressed on the cell surface^31^. Another alternative approach to selecting high IgG secreted hybridoma clones in a semisolid medium is using FITC conjugated anti-mouse IgG antibody combined with an automatic colony picking system. However, there is an expensive technique, and it requires specific instruments.

ICI targeting PD-1 must have at least two required properties: high binding affinity to its target and inhibition of the interaction between PD-1 and PD-L1 molecules. Consequently, we developed a hybridoma screening comprising two steps: primary screening for PD-1 binding activity by ELISA and further screening PD-1/PD-L1 binding blockade activity using duplex high-throughput flow cytometry. Our results demonstrate that 51 mini-pools with high binding activity were successfully selected by primary screening, which narrows down the only highly potent clones for further screening. Using a combination of ELISA screening and high-throughput flow cytometry, we successfully isolated highly potent clones producing anti-PD-1 mAb with PD-1/PD-L1 binding blockade activity.

Collectively, although each of the technique employed for the hybridoma generation and screening in this study has been previously described, we have successfully integrated these technical procedures into a streamlined workflow that can be efficiently executed. Improvement in each step along the whole workflow of the anti-PD-1 mAb generation, i.e., immunization, hybridoma generation, semi-solid clonal selection, and duplex high-throughput screening, ultimately provides five potent chimeric anti-PD-1 candidate prototypes. The xCUSB0103 chimeric lead prototype exhibited high binding affinity, PD-1/PD-L1 binding blockade activity, and T cell-stimulating ability in vitro, comparable to OPDIVO. Further development will be required to construct a humanized anti-PD-1 based on this lead prototype in order to further increase its properties, e.g., reduce immunogenicity and improve binding affinity. Finally, this lead prototype could potentially be developed as an ICI in various formats for cancer immunotherapy.

## Methods

### Mice and cell lines

This study was approved by the Chulalongkorn University Animal Care and Use Protocol (CU-ACUP) ethics committee (protocol number 05/2561). All animal experiments were performed in accordance with the CU-ACUP and ARRIVE (https://arriveguidelines.org) guidelines.

Six- to eight-week-old female BALB/c and ICR mice were purchased from Nomura Siam International Co., Ltd. (Bangkok, Thailand). The P3/NSI/1-Ag4-1 (NS-1) myeloma cell line was purchased from American Type Culture Collection (ATCC). hPD-1-expressing Jurkat effector cells and aAPC/hPD-L1-expressing CHO-K1 cells were purchased from Promega (J1252). hPD-1-expressing CHO-K1 cells were purchased from GenScript (M00529). Expi293F cells were purchased from Gibco (A14635).

The NS-1 myeloma cells were cultured in complete medium containing 15% FBS (Gibco, 10270106), 1 mM Na-pyruvate (Gibco, 11360070), 1× nonessential amino acids (Gibco, 11140050), 2 mM GlutaMax-I (Gibco, 35050061), 1× Pen/Strep (Gibco, 15140122), 50 µg/ml gentamycin (Gibco, 15710064), and 1× beta-mercaptoethanol (Gibco, 21985023) in DMEM (HyClone, SH30022) at 37°C in a 5% CO_2_ humidified incubator. Mouse hybridoma cells were cultured in complete medium without beta-mercaptoethanol. The hPD-1-expressing Jurkat effector cells and aAPC/hPD-L1-expressing CHO-K1 cells were cultured and maintained according to the technical manual recommendation. hPD-1-expressing CHO-K1 cells were cultured in F12K medium (Gibco, 21127022) containing 10% FBS.

### Mouse immunization

BALB/c or ICR mice were immunized (4 mice per strain) with either recombinant hPD-1 Fc chimeric proteins, protein group (R&D System, 1086-PD), or hPD-1-expressing CHO-K1 cells, cell group (GenScript, M00529).

For hPD-1 Fc chimeric proteins, the antigen was emulsified in AdjuLite Freund's complete adjuvant (Pacific Immunology, A5001) at a ratio of 1:1 (v/v) for the first immunization (50 µg/150 µl/mouse), while following boosters, the antigen was emulsified in AdjuLite Freund's incomplete adjuvant (Pacific Immunology, A5002) at 25 µg/150 µl/mouse. The emulsion mixture was injected into 3 sites via subcutaneous (SC) injection at the neck (50 µl) and intramuscular (IM) injection at the left and right thighs (50 µl/site). For hPD-1-expressing CHO-K1 cells, antigen dissolved in sterile PBS buffer (1×10^7^ cells/ 150 µl) was injected via intraperitoneal (IP) injection. Final immunization was performed by injecting both antigens dissolved in sterile PBS buffer via the IP (150 µl).

Immunization was performed every 2-week intervals for the cell group and every 4-week intervals for the protein group for 16 weeks. Both antigens dissolved in sterile PBS buffer were injected (150 µl) via intraperitoneal (IP) injection for the final immunization. Blood was collected after 16 weeks of immunization to determine the hPD-1-specific mouse IgG titer. The highest IgG titer mouse immunized with each antigen was selected for final immunization, and their spleen was collected for hybridoma generation 4 days later.

### Splenectomy and cell electrofusion

The selected mice were anesthetized, and splenectomy was performed using an aseptic technique. Splenocytes were filtered through a 100 µm cell strainer (Falcon, 352360) in a sterile 50 mL conical tube, followed by washing 3 times with serum-free medium (360×*g* at 25°C for 7 min). Splenocytes and myeloma cells were counted using a Countess 3 FL automated cell counter (Invitrogen). Then, splenocytes and myeloma cells were mixed at a ratio of 2:1. The cell mixture was washed 3 times with 10 ml sterile DPBS buffer (Gibco, 14040–133), followed by 3 times 10 ml sterile BTX fusion buffer. The cell mixture was dissolved in ice-cold BTX fusion buffer (5×10^7^ cells/ml) and transferred into electrocuvettes (400 µl/cuvette). Electrofusion was performed using the BTX ECM 2001 machine. Fused cells were recovered in ClonaCell-HY medium C (STEMCELL, 03803) at 37°C in a 5% CO_2_ humidified incubator overnight.

### Hybridoma cloning and selection

A semisolid medium containing HAT (hypoxanthine, aminopterin, and thymidine) was used for hybridoma selection and cloning. Briefly, recovered cells were harvested (360×*g* at 25 °C for 7 min) and reconstituted (2×10^7^ cells/ml) in ClonaCell-HY medium C (STEMCELL, 03803). Cell solutions were then gently mixed with semisolid ClonaCell-HY medium D (STEMCELL, 03804) at a ratio of 1:9 (v/v) according to the ClonaCell-HY technical manual instructions. The cell mixture was plated (2.5 ml/well) into a 6-well cell culture plate (Nunc, 140,675) and incubated at 37°C in a 5% CO_2_ humidified incubator for 7–10 days. Four to six hybridoma colonies were randomly picked by pipetting and transferred to each well (mini-pool) of a 96-well culture plate containing 200 µl of ClonaCell-HY medium E (STEMCELL, 03,805). Mini-pools were cultured for 2 days, and the culture supernatants were taken to determine the hPD-1-specific IgG levels by ELISA.

### ELISAs

ELISA was used to evaluate the PD-1 binding activity as previously described^32^. For ELISA screening, culture supernatant was used as a sample and detected with goat anti-mouse IgG Fcγ-HRP antibody (Jackson ImmunoResearch, 115-035-164) diluted 1:8,000 in 0.05% Tween-20 PBS buffer (PBST). For the mouse PD-1 binding profile, serial dilutions of purified mouse anti-PD-1 mAbs were used. For the chimeric PD-1 binding profile, serial dilutions of purified chimeric anti-PD-1 mAbs were used and detected with goat anti-human IgG Fcγ-HRP antibody (Jackson ImmunoResearch, 109-005-098) diluted 1:10,000 in PBST. The SIGMA*FAST* OPD (Sigma-Aldrich, P9187) substrate solution was used, and the reaction was stopped by adding 1 M H_2_SO_4_. The absorbance was measured at 492 nm by a Cytation 5 cell imaging multi-mode reader (BioTek). Mini-pools providing an A_492_ ELISA signal of more than 1.0 were considered high hPD-1-specific IgG level pools. These mini-pools were further screened for the anti-PD-1 neutralizing antibody (NAb) by flow cytometry.

### Screening and selection of mouse hybridoma producing anti-PD-1 antibodies with PD-1/PD-L1 binding blockade activity by duplex high-throughput flow cytometry

The culture supernatant from each hybridoma mini-pools producing high hPD-1-specific IgG (100 µl) and recombinant hPD-L1 Fc chimeric protein (GenScript, Z03371) (20 µl, 25 µg/ml) was added to a 96-well bottom V plate (Nunc, 249570) containing PD-1-expressing Jurkat effector cells (1.5×10^4^ cells each well) in FPBS buffer (10% FBS in PBS buffer) and gently mixed by pipetting. The plate was incubated at room temperature for 30 min and then centrifuged to harvest the cells (500×*g*, at room temperature for 5 min), and the supernatant was discarded. To detect either mouse IgG or hPD-L1-Fc chimeric protein that bound to the hPD-1-expressing Jurkat cells, the labeled secondary antibody mixture, which was Alexa Fluor 488 goat anti-mouse IgG (Jackson ImmunoResearch, 115-545-062) at 1:200 in FPBS buffer and Alexa Fluor 647 goat anti-human IgG (Jackson ImmunoResearch, 109-605-088) at 1:100 in FPBS buffer, was added to each well (100 µl) and then gently mixed by pipetting. The plate was incubated in the dark at room temperature for 30 min and washed twice with FPBS buffer. Cells were resuspended in FPBS buffer (100 µl). The Intellicyt iQue Screener Plus flow cytometer was used to determine and screen the clones producing anti-PD-1 antibodies that inhibit PD-1/PD-L1 interaction, anti-PD-1 neutralizing antibody (NAb).

Selected hybridoma mini-pools producing anti-PD-1 NAb were subcloned using semisolid medium. A single colony of hybridoma cells was picked by pipetting, transferred to each well of a 96-well plate containing 200 µl ClonaCell-HY medium E and cultured for 2 days. Culture supernatant from each clone was taken to confirm the PD-1 binding and PD-1/PD-L1 binding blockade activities.

### Production and purification of anti-PD-1 monoclonal antibody

Mouse hybridoma clones producing anti-PD-1 mAb were expanded in complete medium for 14 days. Cells were then harvested and cultured in HyClone SFM4MAb medium (Cytiva, SH30513.02) at 37°C in a 5% CO_2_ humidified incubator for 5 days for antibody production. The culture medium was harvested by centrifugation (4000×*g*, at room temperature for 10 min), adjusted into 1× binding buffer (100 mM HEPES, 150 mM NaCl buffer, pH 8.0), and filtered through a 0.45-µm PES membrane. The antibody was purified by a HiTrap Protein A FF column (Cytiva, 17507901) using the ÄKTA pure protein purification system. The captured antibody was eluted with Pierce IgG elution buffer (Thermo Scientific, 21004). Purified antibodies were pooled, concentrated, and buffer-exchanged to PBS buffer (pH 7.4) using the Amicon Ultra-15 (30 kDa) centrifugal filter (Millipore, UFC903024). Purified antibodies were aliquoted and kept at 4°C for short-term storage or − 20°C for long-term storage.

### Generation, production, and purification of chimeric anti-PD-1 monoclonal antibody

Total RNA was extracted from selected hybridoma clones using the RNeasy mini kit (QIAGEN, 74104). Antibody variable regions were amplified using SMART technology as described previously^33^.

The light chain (LC) and heavy chain (HC) genes were grafted into the human kappa (accession number: P01834) and human IgG4 (accession number: P01861) constant regions, respectively, with the mouse signal peptide (GenBank: AAA51634.1) at the N terminus. Both antibody genes were separately cloned into the pcDNA3.4 expression vector (GenScript). Small-scale transient expression was performed according to the Expi293 expression system user guide. Briefly, 15 µg of sterile transfection grade expression vectors (LC: HC ratio of 2:1) were mixed with diluted ExpiFectamine 293. The mixture solution was added to 15 ml of Expi293F cells (3×10^6^ cells/ml) and incubated at 37°C with shaking in 8% CO_2_ for 7 days. The culture medium was harvested, and the antibody was purified as described above.

### PD-1/PD-L1 binding blockade assay

The PD-1/PD-L1 blockade bioassay (Promega, J1252) was performed to evaluate the PD-1/PD-L1 binding blockade activity of the antibodies. In brief, adherent PD-L1 aAPC/CHO-K1 cells (4×10^5^ cells/ml, 100 µl each well) were added to the inner 60 wells of a 96-well flat, white bottom assay plate. The plates were incubated at 37°C in 5% CO_2_ overnight (16–20 h). On the assay day, the medium was aspirated from the inner 60-well plates. The assay plate was supplemented with serial dilutions of antibody samples (40 µl/well) and Jurkat/PD-1 effector cells (1.25×10^6^ cells/ml, 40 µl/well) and then incubated at 37°C in 5% CO_2_ for 6 h. The assay plate was allowed to stand at room temperature for 15 min, and then the Bio-Glo substrate reagent (Promega, G7940) was added to 80 µl of each well and incubated in the dark at room temperature for 5 min. Luminescence signals were measured using the Cytation 5 cell imaging multimode reader and reported as a relative light unit (RLU). The IC_50_ was calculated using GraphPad Prism software.

### PD-1 binding kinetics by SPR

The Biacore T200 instrument, equipped with a Protein G sensor chip (GE Healthcare, 10258853), was used to determine binding kinetics between antibodies and hPD-1 protein. For the mouse antibodies, goat anti-mouse IgG1 (Jackson ImmunoResearch, 115,005,205) at 10 µg/ml was injected into an individual flow cell of a protein G sensor chip (30 µl/min for 60 s), followed by each mouse anti-PD-1 antibody (1 µg/ml) with a flow rate of 30 µl/min for 60 s. For the chimeric antibodies, each chimeric anti-PD-1 antibody (1 µg/ml) was injected into an individual flow cell of a protein G sensor chip with a flow rate of 30 µl/min for 60 s. A single-cycle binding kinetics analysis was performed by sequentially injecting recombinant hPD-1 His tag protein (R&D Systems, 8986-PD) at various concentrations (1.5625, 3.125, 6.25, 12.5, and 25 nM) with interval cycles of association time for 60 s and dissociation time for 120 s. HBS-EP^+^ buffer (GE Healthcare, BR-100669) was used as a buffer blank and diluent for all antibody and protein preparations. The uncoated reference cell and buffer blank signals were subtracted from the sensorgrams. Biacore T200 evaluation software (version 3.1) was used to calculate the association constant (*k*_*on*_), dissociation constant (*k*_*off*_), and equilibrium constant (*K*_*D*_) with a 1:1 Langmuir binding model curve fitting.

### Mixed lymphocyte reaction (MLR) assay

The two-way MLR assay was conducted by GenScript (Nanjing GenScript Biotechnology Co. Ltd.) in accordance with relevant guidelines and regulations. Human PBMCs were collected from healthy donors, with informed consent obtained from all donors. Dendritic cells (DCs) were used as antigen-presenting cells (APCs), and CD4^+^ T cells were used as effector cells at a ratio of 1:5. Serial dilutions of antibody samples (starting from 10 μg/ml) were added to the assay plate containing effector cells. The derived DCs were then added to the plate, gently mixed to initiate the reaction, and cocultured at 37°C in 5% CO_2_ for 3 days. The secretion levels of human interleukin 2 (IL-2) and interferon-γ (IFN-γ) were quantified using a Human IL-2 HTRF kit (Cisbio, 64IL2PEB) and Human IFN-γ HTRF kit (Cisbio, 62IFNPEC), respectively. Commercial anti-PD-1 antibodies (KEYTRUDA and OPDIVO) were used as a positive control, while human IgG4 isotype control was used as a negative control. The EC_50_ value was calculated using GraphPad Prism software.

## Data Availability

All data generated or analyzed during this study are included in this published article.

## Abbreviations

x(prefix): Chimeric
h(prefix): Human
ICI: Immune checkpoint inhibitor
IFN-γ: Interferon gamma
IL-2: Interleukin-2
IM: Intramuscular administration
IP: Intraperitoneal administration
MLR: Mixed lymphocyte reaction
mAb: Monoclonal antibody
NAb: Neutralizing antibody; antibody that inhibit the PD-1/PD-L1 interaction
PD-L1: Programmed cell death ligand 1
PD-1: Programmed cell death protein 1
SC: Subcutaneous administration
